# Income growth, employment structure transition and the rise of modern markets: The impact of urbanization on residents’ consumption of dairy products in China

**DOI:** 10.1371/journal.pone.0267006

**Published:** 2022-04-15

**Authors:** Rongzhu Cheng, Qianqian Wang, Longbao Wei

**Affiliations:** 1 China Academy for Rural Development, Zhejiang University, Hangzhou, China; 2 LICOS Centre for Institution and Economic Performance, KU Leuven, Leuven, Belgium; 3 School of Economics and Management, Zhejiang Sci-Tech University, Hangzhou, China; Institute for Advanced Sustainability Studies, GERMANY

## Abstract

In modern society, dairy products have become increasingly important in our diet because of changes in consumption patterns due to urbanization. However, Chinese residents’ dairy consumption remains at a relatively low level, with great potential for growth. Exploring the main determinants of dairy consumption and their effect mechanisms not only helps to improve the health status of residents, but also has important policy implications for the development of China’s dairy industry. Based on the data of China Health and Nutrition Survey (CHNS) from 1989 to 2011, this study empirically analyzes the impact of urbanization on residents’ dairy consumption. The results indicate that urbanization could significantly promote residents’ consumption of dairy products and the effect is higher in areas with low urbanization levels and in midwestern regions than in areas with high urbanization levels and in midwestern regions. From the perspective of effect mechanism, income growth, employment structure transition and the rise of modern markets are three important mediating paths. Additionally, the results imply that in areas with low urbanization levels, income growth and the rise of modern markets are the main significant mediators; while in areas with high urbanization levels, employment structure transition is a significant mediator. Moreover, in midwestern regions, income growth is a significant mediator, and employment structure transition is a significant mediator in all regions. These findings have practical implications for understanding the relationship between urbanization and residents’ food consumption and for further promoting residents’ dairy consumption and the development of China’s dairy industry.

## Introduction

Since the reform and opening up, China’s urbanization rate has continued to increase, from the 17.92% in 1978 to 51.27% in 2011 and more than 64% in 2021. In the process of urbanization, residents’ food consumption patterns have undergone significant changes, which are mainly reflected in the gradual decline in the consumption of cereal grains and continuous increase in the consumption of animal products [1,2].

As animal-derived foods are rich in nutrients, dairy products have become an important part of residents’ food consumption. However, the dairy consumption of Chinese residents remains at a relatively low level. Residents’ dairy consumption expenditure accounts for only a small percentage of the total food expenditure, and the per capita consumption of dairy products is only one-third of the world average, which indicates great potential for further growth. Therefore, exploring the main determinants of dairy consumption and their effect mechanisms not only helps to improve the health status of residents, but also has important policy implications for the development of China’s dairy industry.

Existing empirical research on residents’ dairy consumption mainly focuses on residents’ consumption characteristics and preferences for dairy products [3–7], the factors affecting dairy consumption [8–14], prospects for the future consumption of dairy products [15] and the impact of the 2008 milk scandal on residents’ dairy consumption behavior [16,17]. However, few studies directly test the relationship between urbanization and residents’ dairy consumption; so that is one main objective in this study. In addition, most existing studies residents’ dairy consumption only consider urban residents [4,10,12,14,16], but urbanization is a dynamic process, which is not a simple binary transition; accordingly, this study accounts for rural residents as well.

Main aim of this study is to analyze the relationship between urbanization and residents’ consumption of dairy products in China using the data collected from 1989–2011 CHNS surveys. We first calculate residents’ average daily dairy consumption amount, urbanization rate, and the main key variables in each community. Subsequently, we take both urban and rural residents as research samples to explore the impact of urbanization on residents’ dairy consumption. Next, we use a parallel multiple mediator model to analyze the impact paths and mechanisms of urbanization on residents’ dairy consumption, and find that income growth, employment structure transition and the rise of modern markets are three significant mediators. Additionally, we divide the samples into community groups with different urbanization levels and groups from different regions to explore the heterogeneity. Last, we use the percentile bootstrap method to check the robustness of the models and instrumental variable method to avoid potential endogeneity problems.

The remaining contexts are organized as follows: Section 2 reviews relevant literature and introduces the study area and proposes the research hypotheses. Section 3 introduces the data and models used in this study. Section 4 conducts the empirical analysis, including the regression of the baseline models, heterogeneity analysis, robustness test and potential endogeneity solving. Section 5 provides the conclusions, discussions and directions for future works.

## Literature review and research hypotheses

According to extant literature, urbanization mainly affects residents’ dairy consumption through three paths: income growth, employment structure transition, and the rise of modern markets.

### Income growth

In most of the studies on dairy consumption, income is found to be an important factor [3,7,10–15,18]. During the urbanization process, a large number of rural laborers migrate to urban areas. Urban local residents can obtain more income through the division of labor and cooperation with rural migrants [19,20]. Additionally, when the urbanization rate reaches a certain level, an improvement in urbanization can bridge the urban-rural income gap [21].

Keynes’s consumption theory believes that people’s consumption is a function of income level. The increase in income will directly enhance residents’ food consumption capacity [22] and reduce the proportion of staple foods in overall diets [23], which will undoubtedly help increase the consumption level of residents’ dairy products.

Accordingly, the improvement of urbanization promotes residents’ income levels, and income growth further promotes residents’ consumption of dairy products. Therefore, we consider income growth as an important mediation effect.

### Employment structure transition

**Fig 1:**
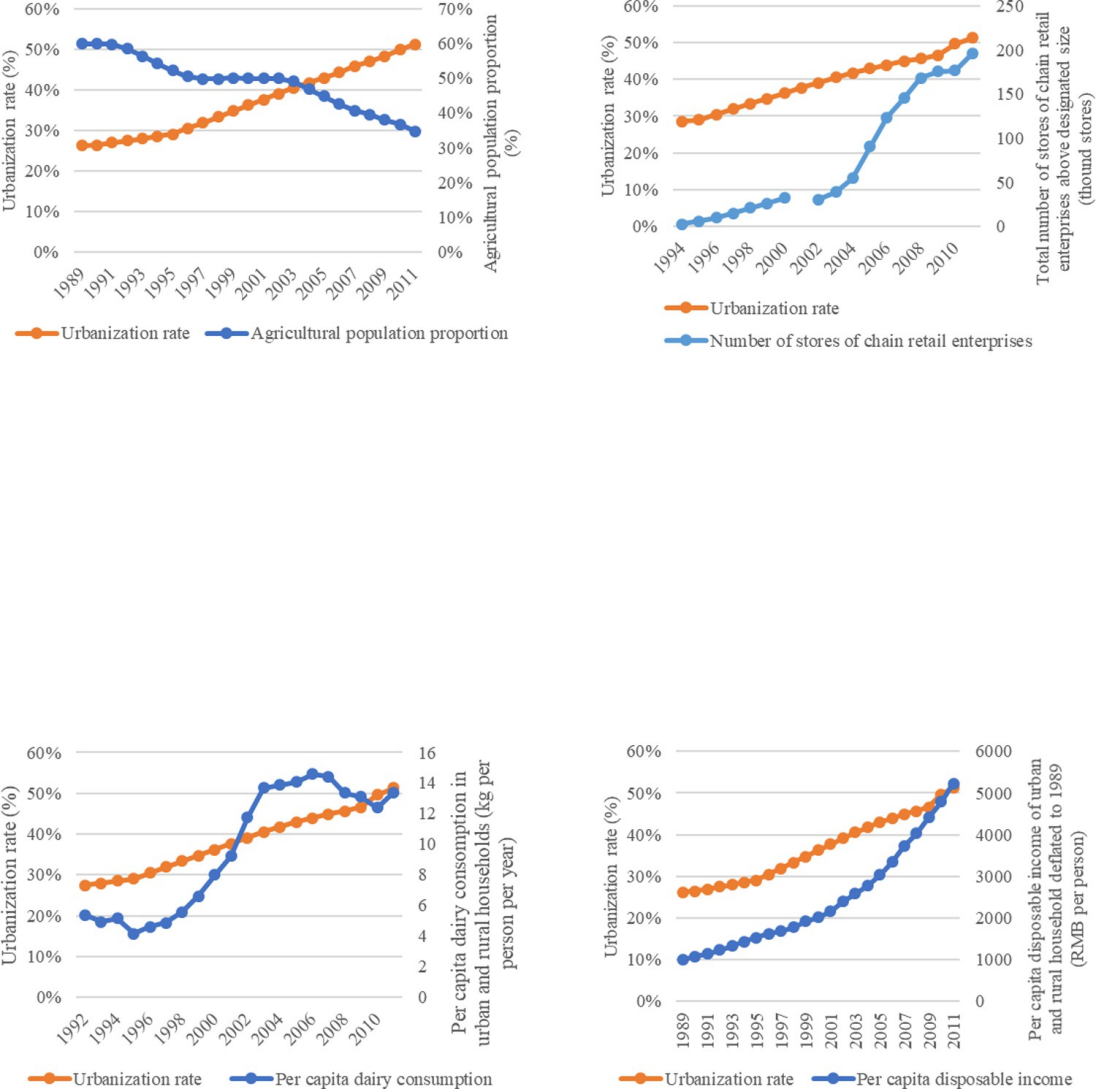
Urbanization and statistics related to residents’ consumption of dairy products. Note: Data source: *China Statistical Yearbook*, *China Dairy Yearbook*. The chain retail enterprises above designated size contains chain retail enterprises with 60 employees or more and annual sales of 5 million RMB or more.

In the process of urbanization and economic development, populations transfer at both spatial and industrial levels. In addition to the increase in urban population, residents’ employment structure gradually shifts from agricultural sectors (See Fig 1), which mainly comprise hard manual labor, to non-agricultural sectors (e.g., non-manual service industries) [24]. People who engage in non-agricultural sectors consume less physical energy and thus calorie inputs than that in agricultural sectors, and this changes their food consumption habits to some extent, leading to a decrease in the demand for staple food [1,25], and a potential increase in the demand for more nutritional foods such as dairy products.

Accordingly, the increase of urbanization level reduces the participation rate in agricultural sectors, and the reduction of manual labors in agriculture sectors, in turn, boosts residents’ demand for dairy products. Therefore, we consider employment structure transition as an important mediation effect.

### The rise of retail terminals represented by supermarkets

The process of population gathering in cities also promotes the continuous concentration of retail terminals in cities. Convenient consumption channels will promote the purchase and consumption of dairy products in cities with high urbanization and development level.

In 2011, the total number of chain retail stores in China was 195.8 thousand, approximately tenfold that of the late 1990s, and the 1990s and beginning of the 21st century were periods of rapid urbanization in China (See Fig 1). Areas with high urbanization level have larger and more modern supermarkets [26]. Researchers find that supermarkets and modern retail stores have a significant positive impact on residents’ purchases of dairy products [3,10]. Supermarkets can capitalize on large consumers inflows, rich product categories, and cold chain guarantee when competing with traditional retail terminals [27]. Some dairy varieties, such as the pasteurized milk, have relatively high transaction costs (including the cost of frequent purchases and storage costs), and the increasing level of market convenience makes the consumption of these dairy products possible [28]. Moreover, the rise of modern markets has helped dairy products from western China enter the eastern market and broken the monopoly of local dairy processing companies.

Accordingly, the process of urbanization promotes the rise of modern markets, and the development of retail network system further helps promote residents’ dairy consumption. Therefore, we consider employment structure transition as an important mediation effect.

Fig 1 shows the urbanization process, residents’ dairy consumption and the indicators reflecting the three potential impact paths described above, from the 1990s to the beginning of the 21st century. Evidently, residents’ income level and the development of modern chain retail enterprises have a common trend with the urbanization process and residents’ dairy consumption rise, while the employment in agricultural sectors has an opposite trend.

Based on the above analysis, this study proposes the following hypotheses:

H1: Income growth plays a mediating role in the impact of urbanization on residents’ dairy consumption. Specifically, the improvement of urbanization promotes residents’ income levels, and income growth further promotes residents’ consumption of dairy products.
H2: Employment structure transition plays a mediating role in the impact of urbanization on residents’ dairy consumption. Specifically, the improvement of urbanization reduces the participation rate in agricultural sectors, and the transition of employment structure boosts residents’ demand for dairy products.
H3: The rise of modern markets plays a mediating role in the impact of urbanization on residents’ dairy consumption. Specifically, the improvement of urbanization promotes the rise of modern markets, and the rise of modern markets further promotes residents’ dairy consumption.

**Fig 2:**
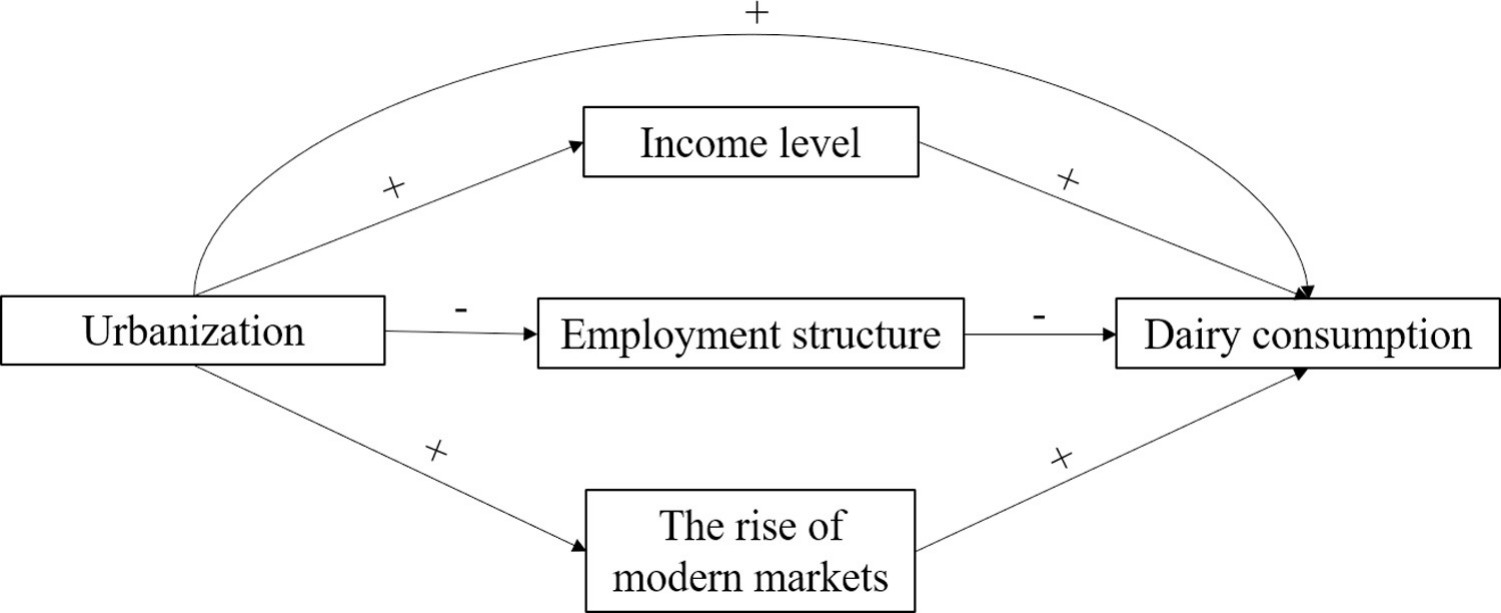
The relationships and influence paths between the variables.

The relationships and influence paths between the variables in the hypotheses are depicted in Fig 2.

## Data and models

### Data

The data used in this study are obtained from the China Health and Nutrition Survey (CHNS). The samples in the CHNS comprise populations from provinces in the east, northeast, central and west regions of China, which vary substantially in geography, economic development, public resources, and health indicators; thus, it is nationally representative. For the sample, we select the full period data of 1989, 1991, 1993, 1997, 2000, 2004, 2006, 2009 and 2011. The year 2015 is omitted because the data from the diet survey, which is the important source for our dependent variable, has not been released to the public. All variables in this study are obtained from community-level data or have been aggregated to the community level.

### Variables

#### Dependent variable

The dependent variable is the residents’ average daily consumption of dairy products. The CHNS nutrition surveys include the household food survey and the individual diet survey. Both surveys are conducted on three randomly chosen consecutive days of a week at the same time. The household food survey adopts the food inventory method, that is, it records the daily purchasing amount and discarding amount of each food item and the number of people dining in the household in detail. The daily consumption of each food item per person is calculated from the changes in food inventory. Differently, the individual diet survey adopts the 24-hour Dietary Recall. This method requires the respondent to recall the food type, quantity, eating time, dining place, and preparation method within the last 24 hours. Compared with the individual diet survey, the household food survey might be biased if there are guests eating at respondent’s home during the survey period; furthermore, the household food survey does not consider the cases of dining out. However, with the development of the economy and the increase of residents’ income, dining out has become increasingly frequent for Chinese residents. Therefore, to obtain more precise consumption data, we finally choose the dataset from the individual diet survey based on the 24-hour Dietary Recall.

To identify the dairy products in the CHNS individual diet survey, we refer to the book *China Food Composition*. The data of 1989, 1991 and 1993 refers to the edition published in 1989; the data of 1997 and 2000 refers to the edition published in 1993; the data of 2004, 2006, 2009 and 2011 refers to the edition published in 2004. We calculate the average consumption amount of each person per day during the three survey days, and then aggregate to the community-level. The units from different years are unified to grams (g).

#### Key independent variable

The key independent variable in this study is the urbanization level. For the purposes of this study, it is calculated as the proportion of permanent residents with urban *hukou* i in the total community population. The individuals who have been abroad, moved out to other cities or lived in the local community for less than six months each year are not considered in the calculation.

#### Mediating variables

Based on the literature review, this study selects three mediating variables to explore the ways in which urbanization affects residents’ dairy consumption: income level, employment structure and the rise of modern markets. In this study, Income level is the per capita annual net income at the community level in logarithmic form. Employment structure is defined as the proportion of people engaged in the agriculture sector among the total labor force. We employ the market component scores provided by the CHNS, which considers the types of markets available, distance to the markets in or near the local community, and number of days these markets are open, to evaluate the local development of modern markets in each community [29]. The higher the score a community receives, the better development of the modern markets in the community.

#### Control variables

This study selects the purchasing power, production value of cow products, proportion of the elderly and children among the total population, education level, and 2008 milk scandal as the control variables.

Purchasing power significantly affects residents’ consumption expectations for food [30]. The decline in purchasing power has a negative impact on residents’ dairy consumption. In this study, purchasing power is indicated by the Consumer Price Index.

It is difficult to obtain the dairy output data for each community from the CHNS survey. Instead, we consider the total value of the products produced from the third livestock type (i.e., cows and horses).

The increase in the number of elderly people and young children might enhance the demand for dairy products. In this study, the proportion of the elderly and children is calculated by measuring the proportion of residents in community who are older than 65 years and younger than 6 years.

The improvement of education level will improve residents’ cognition and consumption preferences for dairy products. In this study, we calculate the residents’ average years of education for each community.

Food safety incidents, such as the melamine incident that occurred in 2008, have a direct inhibitory effect on the consumption of dairy products. Therefore, a dummy variable “milk scandal” is added to the control, for which the years before and after 2008 are coded as 0 and 1, respectively.

Table 1 provides the definitions of the variables.

**Table 1:** Definition of variables.

Variables		Code	Definition	unit
Dependent variable	Dairy consumption	Q	Residents’ average daily consumption of dairy products	gram
Independent variable	Urbanization level	UR	The proportion of permanent residents with urban hukou in total population	/
Mediating variable	Income level	IL	Log (per capita annual net income)	/
	Employment structure	ES	The proportion of people engaged in agriculture sector among the total labor force	%
	Rise of modern markets	RM	The evaluation of development of local retail markets	Score 0–10
Control variable	Purchasing power	Contr_1_	Consumer Price Index	/
	Production value of cow products	Contr_2_	The value of products produced from the third livestock type	CNY
	Proportion of the elderly	Contr_3_	The proportion of residents older than 65	/
	Proportion of children	Contr_4_	The proportion of residents under 6	/
	Education level	Contr_5_	Residents’ average years of education	year
	Milk scandal	Contr_6_	Year before or after 2008	0/1

### Models

First, we construct the following model:

$$Q_{it}=\alpha_{0}+\alpha_{1}{UR}_{it}+\sum_{2}^{k}\alpha_{k}{Contr}_{kit}+{Year}_{t}+\varepsilon_{1it}$$ (1)

where *Q*_*it*_ is the average amount of dairy products consumed per person per day in community *i* in year *t*, *UR*_*it*_ is the proportion of permanent residents with urban *hukou* in the total population of community *i* in year *t*, *Contr*_*kit*_ are *k* controlled variables, *Year*_*t*_ is the time effect, and *ε*_*it*_ is the random disturbance term.

Additionally, the following models on mediators are used to construct the whole mediating effect model:

$$M_{it}=\beta_{m0}+\beta_{m1}{UR}_{it}+\sum_{2}^{k}\beta_{mk}{Contr}_{kit}+{Year}_{t}+\varepsilon_{m2it}$$ (2)

$$Q_{it}=\gamma_{m0}+\gamma_{m1}{UR}_{it}+\gamma_{m2}M_{it}+\sum_{3}^{k}\gamma_{mk}{Contr}_{kit}+{Year}_{t}+\varepsilon_{m3it}$$ (3)

where *M*_*it*_ are *m* mediating variables (m = 1: income level, 2: employment structure, and 3: the rise of modern markets), and *Contr*_*kit*_ are the same controlled variables as in Model 1.

The procedure for testing the mediating effect is as follows [31,32]: First, the direct effect model of the impact of urbanization level and control variables on residents’ dairy consumption is regressed (Model 1). If coefficient *α*_1_ is significant, there is a mediating effect; otherwise, there is a suppressing effect because different mediators may have opposite effects. Second, the key independent variable, urbanization level, is regressed on three mediating variables: income level, employment structure and the rise of modern markets (Model 2). Third, the three mediating variables, key independent variable and control variables are added to the whole regression (Model 3). If the coefficients of both *β*_*m*1_ and *γ*_*m*2_ are significant, the indirect effect is significant, and the analysis proceeds to step 5; if at least one of the coefficients is not significant, the analysis proceeds to Step 4. Fourth, the bootstrap method needs to be used to test whether the interaction term (*β*_*m*1_*γ*_*m*2_) is significant. If the interaction term is significant, the indirect effect is significant, and the analysis proceeds to step 5; whereas if it is not significant, the analysis is terminated at this step. Fifth, the coefficient of urbanization level *γ*_*m*1_ in Model 3 is tested. If *γ*_*m*1_ is significant, the direct effect is significant, and the analysis proceeds to the final step. If *γ*_*m*1_ is not significant, then no direct effect exists, indicating that there is only a mediating effect. Lastly, the signs of the interaction term *β*_*m*1_*γ*_*m*2_ and coefficient *γ*_*m*1_ are compared: if the signs are the same, there is a complementary mediating effect; if the signs are different, there is a competitive effect. This procedure is proposed by Zhao et al. (2010), which is improved on the basis of Baron-Kenny procedure and considers the bootstrap test as a supplement reference.

### Statistical analysis

Table 2 presents the descriptive statistics for each variable from 1989 to 2011. A total of 1,036 observations are made. It can be seen that the average residents’ dairy consumption is 171.06 grams per day, and the average urbanization level is around 0.58. The urbanization level in the survey is slightly higher than the actual urbanization rate. One reason is that the number of urban samples (161 communities) is higher than that of rural samples (129 communities). For years with similar numbers of urban and rural communities, such as 2011, the surveyed urbanization level (average of 0.577 in surveyed provinces) is similar to the actual urbanization rate (average of 0.565 in the same provinces). As for the mediating variable, around 31% of residents engage in the agricultural sector on average, and the average modern markets development score is 5.61. As for the control variables, on average, there are about 14% and 2% of the elderly and young children, respectively, in the surveyed communities. Approximately 37.16% of observations are made after the 2008 milk scandal and 62.84% observations are made before 2008.

**Table 2:** Descriptive statistics results.

Variables		Min	Max	Mean	S.D.
Dependent variable	Dairy consumption	1.50	750.00	171.06	84.30
Independent variable	Urbanization level	0.00	1.00	0.58	0.41
Mediating variables	Income level (log)	6.33	11.08	8.94	1.04
	Employment structure	0.00	1.00	0.31	0.38
	Rise of modern markets	0.00	10.00	5.61	3.30
Control variables	Consumer Price Index	0.93	4.10	2.38	0.73
	Production value of cow products	0.00	51200.00	183.25	1908.63
	Proportion of the elderly	0.00	0.58	0.14	0.11
	Proportion of children	0.00	0.27	0.02	0.03
	Education level	2.84	14.18	7.85	2.06
	Milk scandal	Yes (37.16%); No (62.84%)			

## Empirical analysis

### Main results

The empirical analysis is mainly using the statistical software Stata version 14.0. First, we start from the Model 1. This model is used to examine the direct effect of urbanization level on residents’ dairy consumption. In addition to the time fixed effect, we include the province as a fixed effect to control the provincial effect. The empirical results of Model 1 are presented in Column 1 of Table 3. The results show that the coefficient of the key independent variable, urbanization level (*α*_1_), is positive but insignificant. From the literature discussion above, the increase in income level and transition in employment structure may have opposite effects on dairy consumption, so the insignificance of the coefficient is reasonable. This indicates that the increase in urbanization rate is related to an increase in residents’ dairy consumption, but the specific impact mechanism needs to be further explored.

**Table 3:** Regression results of Model 1 and Model 3^a^.

Variables		(1)	(2)	(3)	(4)	(5)
		Q	Q	Q	Q	Q
Independent variable	UR	4.196	0.370	-29.823***	-0.192	-28.579**
		(8.452)	(8.658)	(11.522)	(8.773)	(11.500)
Mediating variables	IL		20.499**			14.549*
			(8.092)			(8.376)
	ES			-49.114***		-39.712***
				(11.294)		(12.070)
	RM				1.341*	0.780
					(0.780)	(0.788)
Control variables	Contr1	-0.976	-3.902	-4.915	-1.853	-6.747
		(11.865)	(11.765)	(12.025)	(11.824)	(11.902)
	Contr2	-0.000	-0.000	-0.000	-0.000	0.000
		(0.001)	(0.001)	(0.001)	(0.001)	(0.001)
	Contr3	23.297	21.167	25.144	25.255	24.417
		(24.681)	(24.602)	(24.460)	(24.669)	(24.487)
	Contr4	-235.081**	-247.515**	-244.850**	-238.083**	-253.552**
		(104.445)	(106.091)	(101.481)	(104.609)	(103.432)
	Contr5	4.452**	2.119	2.711	4.317**	1.310
		(1.834)	(2.129)	(1.793)	(1.838)	(2.075)
	Contr6	92.482***	47.386	103.064***	93.912***	69.863**
		(23.747)	(30.688)	(23.732)	(23.638)	(31.231)
Province fixed effect		Yes	Yes	Yes	Yes	Yes
Time fixed effect		Yes	Yes	Yes	Yes	Yes
R-squared		0.32	0.33	0.33	0.32	0.34
Observations		1036	1036	1036	1036	1036

^a^ *, ** and *** represent that the statistics are significant at the 10%, 5% and 1% levels, respectively.

Then, we further regress urbanization level on the three mediating variables: income level, employment structure and the rise of modern markets (see Table 4). It can be seen that the coefficients of all mediating variables (*β*_11_, *β*_21_, *β*_31_) are significant. This indicates that the increase in urbanization rate is related to a significant increase in residents’ income level, decrease in agricultural engagement, and improvement in the local development of modern markets.

**Table 4:** Regression results of Model 2^a^.

Variables	(1)	(2)	(3)
	IL	ES	RMM
UR	0.187***	-0.693***	3.272***
	(0.042)	(0.027)	(0.383)
Contr1	0.143**	-0.080**	0.653
	(0.062)	(0.038)	(0.615)
Contr2	-0.000	0.000**	-0.000***
	(0.000)	(0.000)	(0.000)
Contr3	0.104	0.038	-1.460
	(0.106)	(0.054)	(1.040)
Contr4	0.607	-0.199	2.239
	(0.460)	(0.278)	(4.031)
Contr5	0.114***	-0.035***	0.101
	(0.009)	(0.004)	(0.072)
Contr6	2.200***	0.215**	-1.066
	(0.122)	(0.085)	(1.220)
Provincial fixed effect	Yes	Yes	Yes
Time fixed effect	Yes	Yes	Yes
R-squared	0.91	0.80	0.26
Observations	1036	1036	1036

^a^ *, ** and *** represent that the statistics are significant at the 10%, 5% and 1% levels, respectively.

Next, the mediating variables are added to the regression on residents’ dairy consumption. Columns 2–4 in Table 3 present the relevant results. Combined with the results in Table 4, the coefficients of the income level, *β*_11_ and *γ*_12_, are both significantly positive, indicating that the development of urbanization enhances residents’ income levels, and the increase in income further promotes the consumption of dairy products. This implies that the “urbanization level–income level–residents’ dairy consumption” path is a significant effect path, and the mediating effect equals 3.83 (20.499×0.187). Similarly, the coefficients of the rise of modern markets, *β*_31_ and *γ*_32_, are both significantly positive, indicating that the “urbanization level–the rise of modern markets–residents’ dairy consumption” path is a significant effect path, and the mediating effect equals 4.39 (1.341×3.272). On the contrary, the coefficients of the employment structure, *β*_21_ and *γ*_22_, are both significantly negative, indicating that the increase of urbanization level reduces the participation rate in agricultural sectors, and the reduction of manual labors in agriculture sectors, in turn, boosts residents’ demand for dairy products. This implies that the “urbanization level–employment structure–residents’ dairy consumption” effect path is also significant, and the mediating effect equals 22.37 ((-30.726)×(-0.728)).

We proceed to check the coefficient *γ*_*m*1_ in Model 3. Table 3 shows that both the value and the significance of coefficient *γ*_*m*1_ decrease after the mediating variables are added to the model. Specifically, the coefficient *γ*_21_ decreases to a significant negative value with an opposite sign to *β*_11_*γ*_12_, indicating a competitive mediating effect for the “urbanization level–employment structure–residents’ dairy consumption” path. The coefficients *γ*_11_ and *γ*_31_ become insignificant, indicating an indirect-only mediation for the “urbanization level–income level–residents’ dairy consumption” path and the “urbanization level–the rise of modern markets–residents’ dairy consumption” paths.

Accordingly, the empirical analysis supports all the hypothesis put forward when considering the full-size sample.

### Heterogeneity analysis

The impact of urbanization on residents’ dairy consumption and its effect mechanisms may differ between areas with different urbanization levels and between different geographic regions of China. To further explore the potential heterogeneity, we divide the samples into high-urbanization-level and low-urbanization-level community groups based on the average urbanization level, as well as community groups from eastern and midwestern regions, and then repeat the above process.

For the insignificant mediation path in regression, the bootstrap test is used to verify its mediation effect. The steps of the percentile bootstrap test are as follows [33,34]: First, random repeated sampling with replacement is implemented based on the original sample to create a new sample. Then, the equations for the dependent variable, with and without mediating variables, are estimated for each bootstrap sample, allowing estimation of ${\hat{\beta }}_{m1},\;{\hat{\gamma }}_{m2}$, and ${\hat{\beta }}_{m1}{\hat{\gamma }}_{m2}$. The above process is repeated B times (usually, B = 5000 times) to obtain the estimation of B mediating effects. After that, the B mediating effects are sorted from small to large and yield sequence C. Finally, the 2.5^th^ and 97.5^th^ percentiles of sequence C are adopted to estimate the 95% confidence interval of the mediating effect. If the confidence interval does not contain the value 0, the mediating effect is significant; otherwise, it is not significant. We use the statistical software SPSS version 18.0 with the Process plug-in version 3.2 written by Andrew F. Hayes to complete the test.

Table 5 presents the results of each mediating path for community groups with different urbanization groups. The results show that in the low-urbanization-level group, “urbanization level–income level–residents’ dairy consumption” path and “urbanization level–rise of modern markets–residents’ dairy consumption” path are the main mediation paths; while in the high-urbanization-level group, “urbanization level–employment structure–residents’ dairy consumption” path is the main mediating path. When in the stage of low urbanization, the income level of residents is relatively low, and residents have limited access to various dairy products; therefore, the increase in income and rise in modern markets represented by supermarkets have a greater impact on the expansion of sales radius and the increase in residents’ consumption of dairy products. With the advancement of urbanization, a large number of laborers move out from agriculture sectors, and their consumption habits also change; then, the role of income levels and modern markets will decline. Therefore, to increase residents’ dairy consumption, more attention should be paid to the improvement of residents’ income level and ensuring residents’ consumption accessibility in the low urbanization level stage. The changes in consumption habits should be based on ensuring residents’ consumption capacity.

**Table 5:** The regression and bootstrap test results of the mediating effects in different urbanization level groups^a^.

	High-urbanization-level communities			Low-urbanization-level communities		
	Regression coefficients of Model 3	Bootstrap tests [95% confidence interval]	Mean (S.D.)	Regression coefficients of Model 3	Bootstrap tests [95% confidence interval]	Mean (S.D.)
IL	6.868	insignificant	9.146	22.835**	/	8.692
	(10.116)	[-6.797, 9.227]	(1.017)	(11.002)		(0.998)
ES	-53.672*	/	0.039	-17.313	insignificant	0.696
	(29.565)		(0.103)	(16.462)	[-26.537, 37.105]	(0.284)
RM	1.205	insignificant	6.791	0.205*	/	3.958
	(1.046)	[-2.851,7.686]	(2.590)	(1.186)		(3.494)
Contr_1-6_	Yes			Yes		
Provincial fixed effect	Yes			Yes		
Time fixed effect	Yes			Yes		
Observations	597			428		

^a^ *, ** and *** in “Regression coefficients of Model 3” represent that the statistics are significant at the 10%, 5% and 1% levels, respectively.

As for the total indirect effect ($\sum_{m=1}^{3}\beta_{m1}\gamma_{m2}$), it is 16.56 for the areas with high urbanization levels and 24.42 for the areas with low urbanization levels. This implies that the impact of urbanization paths on residents’ dairy consumption is stronger at the initial urbanization development stage, and the impact has a marginal decline tendency.

The eastern communities are defined as communities from Beijing, Shanghai, Jiangsu, Shandong, Liaoning, and Heilongjiang provinces, and the midwestern communities are defined as communities from Henan, Hubei, Hunan, Guangxi, Guizhou, and Chongqing provinces. The total indirect effect is 48.80 for the eastern region group, much higher than that for the midwestern region group (24.72). Table 6 presents the results of each mediating path for communities from different regions. The results indicate that the “urbanization level–income level–residents’ dairy consumption” path is a significant mediating path for communities in midwestern regions, but not for those in eastern regions. The average urbanization rate of the communities in midwestern China is 0.55, which is lower than that in eastern regions (0.60), so the result of this path is consistent with the result in low-urbanization-level rate samples. Moreover, “urbanization level–employment structure–residents’ dairy consumption” mediation path is significant in both regions, but plays a larger mediating role in eastern regions. The effect of the “urbanization level–rise of modern markets–residents’ dairy consumption” path does not differ significantly across regions. To increase residents’ dairy consumption, increasing residents’ income is still an effective way in midwestern China, and encouraging the transfer of agricultural manual labor is an effective approach that works in any region.

**Table 6:** The regression and bootstrap test results of the mediating effects in eastern and midwestern regions^a^.

	Communities in eastern regions			Communities in midwestern regions		
	Regression coefficients of Model 3	Bootstrap tests [95% confidence interval]	Mean (S.D.)	Regression coefficients of Model 3	Bootstrap tests [95% confidence interval]	Mean (S.D.)
IL	15.359	insignificant	9.095	25.661**	/	8.781
	(11.012)	[-3.247, 7.933]	(1.023)	(11.714)		(1.031)
ES	-51.017***	/	0.301	-36.608**	/	0.322
	(16.503)		(0.381)	(15.728)		(0.378)
RM	0.992	insignificant	5.227	1.239	insignificant	6.029
	(0.967)	[-4.865, 10.552]	(3.272)	(1.259)	[-5.905, 5.327]	(3.285)
Contr_1-6_	Yes			Yes		
Provincial fixed effect	Yes			Yes		
Time fixed effect	Yes			Yes		
Observations	543			493		

^a^ *, ** and *** in “Regression coefficients” represent that the statistics are significant at the 10%, 5% and 1% levels, respectively; *** in “Boot indirect effect” represents that the mediating effect is significant.

### Robustness test

To check the robustness of the models, we also add the three mediating variables simultaneously to the model in Model 3. The results show that the coefficients of income level and employment structure are still significant, but the coefficient of the rise of modern markets becomes insignificant (Column 5 in Table 3). Therefore, we further use the bootstrap method to test the robustness of the mediating effect through “urbanization level–rise of modern markets–residents’ dairy consumption” path.

Table 7 presents the test results of the mediating effect using the percentile bootstrap method. The results show that the bootstrap confidence interval of the total mediating effect does not contain 0, which means that the total mediating effect, considering all three paths, is significant. Specific to each path, the confidence intervals of “urbanization level–income level–residents’ dairy consumption” path and “urbanization level–employment structure–residents’ dairy consumption” path do not contain 0, and thus, the mediating effects the two paths are significant.

**Table 7:** The test results of the mediating effect using the percentile bootstrap method.

Path	Indirect effect	Boot SE	95% confidence interval	
			LLCI	ULCI
Total	2.141+34.407+1.399→37.947	8.367	21.369	54.283
Income level (IL)	0.221*9.680→2.141	1.741	1.076	5.803
Employment structure (ES)	(-0.715)*(-48.158)→34.407	8.797	16.721	51.603
Rise of modern markets (RM)	3.068*0.456→1.399	2.357	-3.392	5.916
IL/ES	-32.267	9.465	-50.637	-13.471
IL/RM	0.741	2.690	-4.447	6.092
ES/RM	33.008	9.543	13.825	51.958

**Fig 3:**
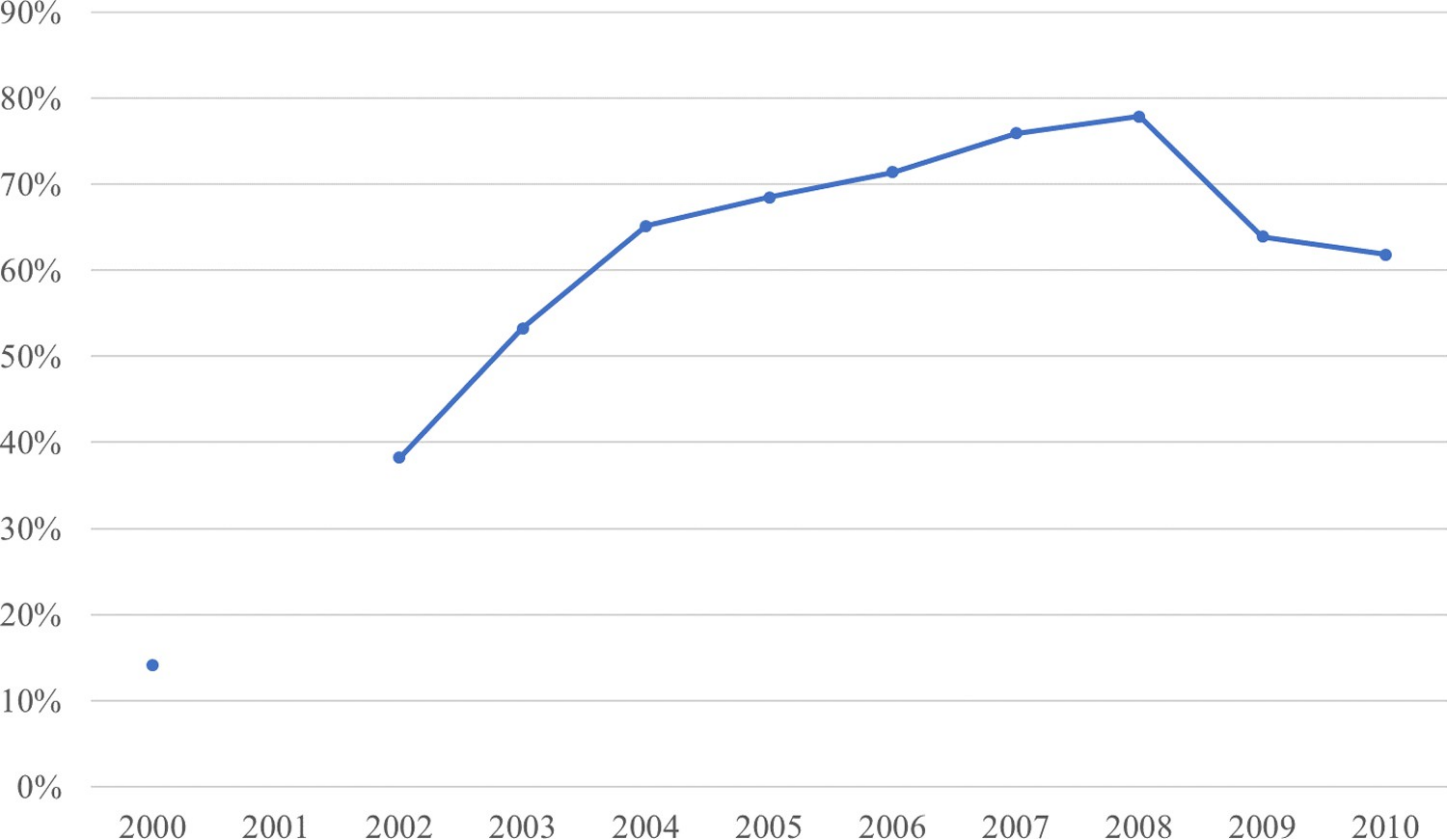
Market share of ultra-high-temperature processing milk 2000, 2002–2010. Note: Data source: China Dairy Yearbook. The market share data of other years has not been revealed by the yearbook.

However, the confidence intervals of the “urbanization level–rise of modern markets–residents’ dairy consumption” path contain 0, indicating that the mediating effect is not significant from the bootstrap test. Therefore, the significance of the mediation of the rise of modern markets is not robust. Considering that the main dairy products consumed by Chinese residents are ultra-high-temperature processed milk (see Fig 3), which is less dependent on storage conditions, the rise of modern markets may have a limited effect on residents’ dairy consumption.

Additionally, the bootstrap test compares the effect sizes of the three mediation paths. The results show that the mediating effect of employment structure is significantly higher than that of income level and the rise of modern markets, while there is no significant difference between income level and the rise of modern markets.

### Potential endogeneity

In order to avoid potential endogeneity problems and non-robust results caused by the estimation method, we adopt the instrumental variable method (IV-2SLS) to further investigate the effect of urbanization on residents’ dairy consumption. We employ the housing condition score and the sanitation score for each community provided by CHNS as the instrumental variables. The housing condition score considers the electricity availability, the possession of indoor tap water and flush toilets, and natural gas usage. The sanitation score considers the proportion of households with treated water and prevalence of households without excreta present outside the home in the community. The two indexes are important indicators of urbanization level, but they are irrelevant with the dairy consumption. Therefore, the two indexes can be the instrumental variables for urbanization level.

Table 8 presents the results of Model 1 estimated by IV-2SLS method. From the first-stage estimation results, it can be seen that both the housing condition and the sanitation condition have a significant positive impact on urbanization level, so there should be no problem with weak instrumental variables, and the Cragg-Donald Wald F statistic further rejects the hypothesis of weak instrumental variables. The coefficient *α*_1_ of both instrumental variables is significantly positive, indicating that the increase in urbanization rate is related to a significant increase in residents’ dairy consumption. Moreover, the significance and signs of other model coefficients are basically consistent with the baseline models. Therefore, the core conclusions of this study still hold.

**Table 8:** The estimation results of Model 1 estimated by IV-2SLS method.

Variables	IV1		IV2	
	The first stage	The second stage	The first stage	The second stage
Urbanization level	/	58.437***	/	53.078***
		(18.536)		(17.139)
Housing condition score	0.076***	/	/	/
	(0.004)			
Sanitation score	/	/	0.059***	/
			(0.004)	
Contr1	0.564***	-33.338**	0.463***	-30.140**
	(0.041)	(16.071)	(0.044)	(14.704)
Contr2	0.000	0.000	0.000	0.000
	(0.001)	(0.001)	(0.001)	(0.001)
Contr3	0.751***	-25.177	0.644***	-20.387
	(0.077)	(25.457)	(0.078)	(26.656)
Contr4	-0.179	-232.927**	0.143	-233.140**
	(0.272)	(102.919)	(0.272)	(102.777)
Contr5	0.062***	-1.732	0.072***	-1.121
	(0.005)	(2.398)	(0.005)	(2.377)
Contr6	-1.573***	172.349***	-1.146***	164.458***
	(0.079)	(33.656)	(0.090)	(31.824)
Provincial fixed effect	Yes	Yes	Yes	Yes
Time fixed effect	Yes	Yes	Yes	Yes
Cragg-Donald Wald F statistic	333.13		313.95	
Observations	1036		1036	

## Conclusions and discussion

This study uses income growth, employment structure transition, and the rise of modern markets as mediating variables, and data from the 1989–2011 CHNS to analyze the impact of urbanization on residents’ dairy consumption in China. The results indicate that urbanization has a significant effect on the promotion of residents’ dairy consumption. Primarily, income growth, employment structure transition and the rise of modern markets mediate the effects of urbanization on residents’ dairy consumption, and all these mediating variables play a positive role. However, the significance of the rise of modern markets is not robust, which can be explained by Chinese consumers’ consumption structure of dairy products. Further, this study explores the heterogeneity in areas with different urbanization levels and in different geographic regions. The results imply that the impact of urbanization on residents’ dairy consumption is larger in areas with high urbanization levels and in eastern regions than areas with low urbanization levels and in midwestern regions. Moreover, income growth is the main significant mediator in areas with low urbanization levels and in midwestern regions, employment structure transition is a significant mediator in areas with high urbanization levels and in all regions, and the rise of modern markets is also a significant mediator in areas with low urbanization levels.

The findings of this study have practical implications for understanding the relationship between urbanization and residents’ food consumption and for further promoting residents’ dairy consumption and the development of China’s dairy industry. First, this study proves that the process of urbanization has a positive impact on residents’ dairy consumption, which indirectly verifies that urbanization will change residents’ food consumption patterns and even their health status. Therefore, to increase residents’ consumption of dairy products and improve their health, the Chinese government needs to promote the urbanization process. Second, from the perspective of the impact path and effect mechanism of urbanization on residents’ dairy consumption, we recommend focusing on improving residents’ income level, employment structure, and the development of modern markets to further increase the consumption level of dairy products. Third, in areas with low urbanization levels, more emphasis should be placed on increasing residents’ disposable income and the development of the retail network, to increase residents’ consumption capacity and accessibility to various dairy products. In areas with high urbanization levels, the government should focus more on improving employment structure to change residents’ consumption habits; this will help better promote residents’ consumption of dairy products. Last, encouraging the transfer of agricultural manual laborers to change their consumption habits is an effective way to increase residents’ dairy consumption in all regions.

In this study, the historical consumption data of dairy products is not continuous, only updated to 2011, and the distribution of consumption data is uneven, which may affect the accuracy of the model establishment. In future work, we will consider using datasets with longer continuous time spans and wider geographical distribution to obtain accurate and precise results.

## Supporting information

S1 File Dataset and explanation. (RAR)

## References

[1] Huang J, David CC. Demand for cereal grains in Asia: the effect of urbanization. Agr Econ-Blackwell. 1993;2(8):107–24. 10.1016/0169-5150(92)90025-T.

[2] Kearney J. Food consumption trends and drivers. Philosophical Transactions of the Royal Society B: Biological Sciences. 2010;365(1554):2793–807. doi: 10.1098/rstb.2010.0149 20713385 PMC2935122

[3] Fuller FH, Beghin JC, Rozelle S. Urban Demand for Dairy Products in China: Evidence from New Survey Data. CARD Working Paper. 2004(04-WP 380).

[4] Fuller F, Beghin J, Rozelle S. Consumption of dairy products in urban China: results from Beijing, Shangai and Guangzhou. The Australian Journal of Agricultural and Resource Economics. 2007;51(4):459–74. 10.1111/j.1467-8489.2007.00379.x.

[5] Wang Z, Mao Y, Gale F. Chinese consumer demand for food safety attributes in milk products. Food Policy. 2008;33(1):27–36. 10.1016/j.foodpol.2007.05.006.

[6] Bernard JC, Bernard DJ. What Is It About Organic Milk An Experimental Analysis. Am J Agr Econ. 2009;91(3):826–36. 10.1111/j.1467-8276.2009.01258.x.

[7] Uzunoz M, Akcay Y. A Case Study of Probit Model Analysis of Factors Affecting Consumption of Packed and Unpacked Milk in Turkey. Economics Research International. 2012;2012:1–8. 10.1155/2012/732583.

[8] Heien DM, Wessells CR. The Demand for Dairy Products: Structure, Prediction, and Decomposition. Am J Agr Econ. 1988;70(2):219–28. 10.2307/1242060.

[9] Cornick J, Cox TL, Gould BW. Fluid milk purchases: a multivariate Tobit analysis. Am J Agr Econ. 1994;76(1):74–82. 10.2307/1243922.

[10] Fuller F, Huang J, Ma H, Rozelle S. Got milk? The rapid rise of China’s dairy sector and its future prospects. Food Policy. 2006;31(3):201–15. 10.1016/j.foodpol.2006.03.002.

[11] Schluep Campo I, Beghin JC. Dairy food consumption, supply, and policy in Japan. Food Policy. 2006;31(3):228–37. 10.1016/j.foodpol.2006.02.009.

[12] Bai J, Wahl TI, McCluskey JJ. Fluid milk consumption in urban Qingdao, China. The Australian Journal of Agricultural and Resource Economics. 2008;52(2):133–47. doi: 10.1111/j.1467-8489.2008.00401.x

[13] Ates HC, Ceylan M. Effects of socio-economic factors on the consumption of milk, yoghurt, and cheese. Brit Food J. 2010;112(2–3):234–50. 10.1108/00070701011029110.

[14] Cheng L, Yin C, Chien H. Demand for milk quantity and safety in urban China: evidence from Beijing and Harbin. Aust J Agr Resour Ec. 2015;59(2):275–87. 10.1111/1467-8489.12065.

[15] Dong F. The outlook for Asian dairy markets: The role of demographics, income, and prices. Food Policy. 2006;31(3):260–71. 10.1016/j.foodpol.2006.02.007.

[16] Zhou Y, Wang E. Urban consumers’ attitudes towards the safety of milk powder after the melamine scandal in 2008 and the factors influencing the attitudes. China Agr Econ Rev. 2011;3(1):101–11. 10.1108/17561371111103589.

[17] Song Y, Yu H, Lv W. Risk analysis of dairy safety incidents in China. Food Control. 2018;92:63–71. 10.1016/j.foodcont.2018.04.007.

[18] Fukase E, Martin W. Who Will Feed China in the 21st Century? Income Growth and Food Demand and Supply in China. J Agr Econ. 2016;67(1):3–23. 10.1111/1477-9552.12117.

[19] Peri G. Immigrants’ Complementarities and Native Wages: Evidence from California. NBER Working Paper No.12956. 2007.

[20] Ottaviano GIP, Peri G. Rethinking the Effects of Immigration on Wages. J Eur Econ Assoc. 2012;10(1):152–97. 10.1111/j.1542-4774.2011.01052.x.

[21] Li Y, Wang X, Zhu Q, Zhao H. Assessing the spatial and temporal differences in the impacts of factor allocation and urbanization on urban–rural income disparity in China, 2004–2010. Habitat Int. 2014;42:76–82. 10.1016/j.habitatint.2013.10.009.

[22] Keynes JM. The general theory of employment, interest and money. Cambridge, United Kingdom: Springer International Publishing; 2018.

[23] Chang X, DeFries RS, Liu L, Davis K. Understanding dietary and staple food transitions in China from multiple scales. Plos One. 2018;13(4):e195775. doi: 10.1371/journal.pone.0195775 29689066 PMC5915834

[24] Kuznets S. Modern Economic Growth: Findings and Reflections. The American economic review. 1973;63(3):247–58.

[25] Huang J. Social Development, Urbanization and Food Consumption. Social Sciences in China. 1999(04):102–16.

[26] Liao C, Tan Y, Wu C, Wang S, Yu C, Cao W, et al. City Level of Income and Urbanization and Availability of Food Stores and Food Service Places in China. Plos One. 2016;11(3):e148745. doi: 10.1371/journal.pone.0148745 26938866 PMC4777357

[27] Hu D, Reardon T, Rozelle S, Timmer P, Wang H. The Emergence of Supermarkets with Chinese Characteristics: Challenges and Opportunities for China’s Agricultural Development. Dev Policy Rev. 2004;22(5):557–86. 10.1111/j.1467-7679.2004.00265.x.

[28] Liu H, Wahl TI, Seale JL, Bai J. Household composition, income, and food-away-from-home expenditure in urban China. Food Policy. 2015;51:97–103. 10.1016/j.foodpol.2014.12.011.

[29] Jones-Smith JC, Popkin BM. Understanding community context and adult health changes in China: Development of an urbanicity scale. Soc Sci Med. 2010;71(8):1436–46. doi: 10.1016/j.socscimed.2010.07.027 20810197 PMC2942954

[30] Rae AN. The effects of expenditure growth and urbanisation on food consumption in East Asia: a note on animal products. Agr Econ-Blackwell. 1998(18):291–9. 10.22004/ag.econ.174423.

[31] Baron RM, Kenny DA. The moderator–mediator variable distinction in social psychological research: Conceptual, strategic, and statistical considerations. J Pers Soc Psychol. 1986;51(6):1173–82. doi: 10.1037//0022-3514.51.6.1173 3806354

[32] Zhao X, Lynch JG, Chen Q. Reconsidering Baron and Kenny: Myths and Truths about Mediation Analysis. J Consum Res. 2010;37(2):197–206. 10.1086/651257.

[33] Hayes AF. Introduction to Mediation Moderation and Conditional Process Analysis: a regression-based approach. New York: the Guilford Press; 2013.

[34] Preacher KJ, Hayes AF. SPSS and SAS procedures for estimating indirect effects in simple mediation models. Behav Res Methods Instrum Comput. 2004;36(4):717–31. doi: 10.3758/bf03206553 15641418

